# P088 Navigating large-vessel giant cell arteritis with steroid psychosis, low IgG and bowel obstruction

**DOI:** 10.1093/rap/rkag113.118

**Published:** 2026-11-03

**Authors:** Hirushi S Jayasekera, Ayman Askari

**Affiliations:** Shrewsbury and Telford Hospitals NHS Trust, Shrewsbury, United Kingdom; The Robert Jones and Agnes Hunt Orthopaedic Hospital, Oswestry, United Kingdom

## Abstract

**Introduction:**

Giant cell arteritis (GCA) with large-vessel vasculitis (LVV) is treated with high-dose glucocorticoids^3^, yet older patients are at risk of steroid-toxicity.^3,4^ Systemic-corticosteroids can cause psychosis in 3-6% of patients^4.5^. Tocilizumab is the only licensed steroid-sparing agent for GCA in the UK^6^; its use is constrained by infection^7^ and gastrointestinal risk^7^.

We report a complex PET-confirmed case of LVV/GCA in a 75-year-old woman with hypogammaglobulinemia, infections and steroid-induced psychosis, where small-bowel-obstruction rendered IL-6 blockade contraindicated. This case illustrates the importance of multidisciplinary decision-making when all standard options carry substantial risks.

**Case description:**

A 75-year-old woman with past anorexia, severe osteoporosis, breast cancer and chronic low IgG was admitted with confusion and behavioural change and recently diagnosed large-vessel vasculitis. She was taking prednisolone 35 mg daily, having previously received 40 mg for one month despite body weight below 40 kg. She reported poor-concentration, personality-change, weight-loss and anorexia, but denied temporal headache, jaw claudication or visual loss. On examination, she was cachectic, drowsy and confused, with a weak left temporal pulse and a swollen right third-MCP. Blood tests showed sodium 117–129 mmol/L, CRP 7–8 mg/L, neutrophilia, platelets 494 ×10^9/L and IgG 4.4 g/L.

Correspondence from the diagnosing center in London showed that eight weeks prior, she had developed unilateral headache with raised inflammatory markers and thrombocytosis. MRI brain and cervical spine were non-specific, although PET-CT demonstrated FDG-avid vertebral, carotid, aortic, subclavian and femoral arteries, consistent with large-vessel GCA. She was commenced a standard prednisolone regimen (40 mg for 3 weeks, then 35 mg for 1 month, 30 mg for 1 month, then 5 mg reductions every 2 weeks to 10 mg).

Her presentation was felt to represent steroid-induced psychosis with overlapping delirium from hyponatraemia and infection, precluding further high-dose glucocorticoids.

During admission she developed aspiration pneumonia and CT-proven mechanical small bowel obstruction with profound hypokalaemia and sepsis, requiring HDU care. Tocilizumab was commissioned as steroid-sparing therapy for GCA but deferred due active infection and bowel obstruction as unacceptable gastrointestinal perforation risk. IVIG was discussed given recurrent infections and low IgG but rejected at national MDT as unlicensed for large-vessel vasculitis with limited supporting evidence. The specialist rheumatology MDT ultimately recommended prednisolone 10 mg daily, cautious weight-based up-titration if required, aggressive infection treatment and psychiatric liaison. Family concerns were discussed. CRP normalised and repeat PET-CT showed no active vasculitis.

**Discussion:**

This case highlights how standard therapy for large-vessel GCA can become relatively unsafe in frail, multimorbid patients. Prolonged high-dose prednisolone (≥35–40 mg) in a very low body weight patient was followed by acute behavioural change and psychosis, emphasising the need for weight-adjusted dosing, shorter high-dose phases and close monitoring for neuropsychiatric adverse effects. Thereafter, confusion was multifactorial, with contributions from hyponatraemia, infection and delirium as well as steroid toxicity, illustrating the diagnostic complexity in vasculitis patients.

Comorbidities also restricted steroid-sparing strategies. Chronic hypogammaglobulinaemia and recurrent infection made conventional immunosuppressants less attractive^9^, while tocilizumab, although commissioned appropriately for steroid-intolerant GCA^10^, had to be deferred because of aspiration pneumonia and small bowel obstruction. In this setting, IL-6 inhibition was felt to carry gastrointestinal perforation risk, according to published safety data^11^.

IVIG was explored as a theoretical option to support low IgG and reduce infection burden while offering immunomodulation^12^, but the national MDT recognised that current evidence supports IVIG primarily for defined antibody-deficiency states or selected vasculitis subsets^13^, not large-vessel GCA, and that it is unlicensed and high-cost in this indication, although it remains a possible option. The pragmatic plan of low-dose prednisolone, psychiatric support and aggressive infection management reflected an evidence-based balance between vasculitis control and treatment-related risk.

For UK practice this case underscores several points: the limited licensed treatment options for LVV/GCA beyond glucocorticoids and tocilizumab; the importance of high-cost drug commissioning pathways; the need to individualise steroid regimens to frailty and body weight; and the central role of multidisciplinary decision-making when biologics are considered.

**Key learning points:**

• High-dose prednisolone in a frail, low-body-weight older patient can precipitate neuropsychiatric toxicity. New behavioural change or confusion after glucocorticoid initiation should prompt immediate consideration of psychosis and early review of dose and tapering strategy.

• Confusion and agitation in vasculitis are often multifactorial. In this case, hyponatraemia, infection, delirium and steroid effects overlapped, so multidisciplinary input (acute medicine, psychiatry, rheumatology) was required to guide safe treatment.

• Chronic low IgG and recurrent infections significantly restrict immunosuppressive options. Although IVIG may benefit defined antibody-deficiency states, there is no current guideline to support using IVIG as primary therapy for large-vessel GCA.

• Tocilizumab is the main licensed steroid-sparing agent for GCA in UK^6^, but active infection and bowel pathology may represent serious contraindications due to perforation concerns and the need to avoid IL-6 in sepsis

• National MDT input was pivotal in this case, supporting a cautious strategy of low-dose prednisolone with psychiatric liaison and close reassessment. This illustrates how high-cost drug commissioning structures can facilitate, but also appropriately limit, biologic use in complex large-vessel vasculitis.

